# Gonococcal PorB: a multifaceted modulator of host immune responses

**DOI:** 10.1016/j.tim.2023.10.002

**Published:** 2024-04

**Authors:** Rebekah A. Jones, Ann E. Jerse, Christoph M. Tang

**Affiliations:** 1 Sir William Dunn School of Pathology, University of Oxford, South Parks Road, Oxford OX1 3RE, UK; 2 Department of Microbiology and Immunology, Uniformed Services University, Bethesda, MD, USA

**Keywords:** *Neisseria gonorrhoeae*, PorB, outer-membrane vesicles, immune responses, vaccine

## Abstract

*Neisseria gonorrhoeae* is a human-specific pathogen responsible for the sexually transmitted infection, gonorrhoea. *N. gonorrhoeae* promotes its survival by manipulating both innate and adaptive immune responses. The most abundant gonococcal outer-membrane protein is PorB, an essential porin that facilitates ion exchange. Importantly, gonococcal PorB has several immunomodulatory properties. To subvert the innate immune response, PorB suppresses killing mechanisms of macrophages and neutrophils, and recruits negative regulators of complement to the gonococcal cell surface. For manipulation of adaptive immune responses, gonococcal PorB suppresses the capability of dendritic cells to stimulate proliferation of T cells. As gonococcal PorB is highly abundant in outer-membrane vesicles, consideration of the immunomodulatory properties of this porin is critical when designing gonococcal vaccines.

## Highlights

The essential gonococcal outer-membrane porin, PorB, manipulates the innate immune response via recruitment of negative regulators of the complement system, and by repressing the killing mechanisms of macrophages and neutrophils.
Gonococcal PorB in its native folded conformation suppresses the capability of dendritic cells to stimulate proliferation of T cells.
Although neisserial PorB is well described, there is conflicting information on the immunomodulatory properties of PorB from different *Neisseria* species.
Studying PorB as protein aggregates or in its native folded state can generate conflicting results, an important consideration for future research.
As PorB is the most abundant protein in gonococcal outer-membrane vesicles, so the immunomodulatory properties of this porin should be carefully considered in developing a successful gonorrhoea vaccine.

## Introduction

The asymmetrical outer-membrane of Gram-negative bacteria is an important barrier to the external environment, and contains outer-membrane proteins (OMPs) needed to transport small hydrophilic molecules, essential nutrients, and ions into the periplasm. Porins are the most abundant family of OMPs, forming trimeric β-barrel pores that allow the passive movement of small solutes into the cell [1]. Importantly, alongside allowing access of essential molecules, bacterial porins have several roles in virulence, through mediating adhesion to and invasion of host cells, activation of the innate and adaptive immune responses, and evasion of host defence mechanisms [1,2].

The genus *Neisseria* contains the human pathogens *Neisseria meningitidis*, a major cause of bacterial meningitis, and *Neisseria gonorrhoeae*, the aetiological agent of the sexually transmitted infection gonorrhoea, alongside numerous commensal species [3]. Porins are highly abundant in the outer membrane of pathogenic *Neisseria* species. *N. meningitidis* expresses two major porins, PorA and PorB, whereas other *Neisseria* species express only one main porin, which is most closely related to PorB [4]. *N. gonorrhoeae* expresses PorB [initially known as Protein I (PI)] from a single locus belonging to either the PorB.IA or PorB.IB class [5]. A *porA* pseudogene is present in the majority of *N. gonorrhoeae* isolates [6], which is not expressed due to mutations in the promoter region and a frameshift mutation in the coding sequence [7].

Gonococcal PorB has defined roles in ion exchange, nutrient acquisition and modulating antibiotic accessibility. Importantly, gonococcal PorB has also been found to subvert immune responses. Given that gonococcal PorB accounts for over 60% of the outer-membrane proteome and is also highly abundant in outer-membrane vesicles (OMVs) [8], the immunomodulatory functions of PorB represent a vital consideration in the development of gonococcal vaccines, the subject of intense recent interest. The purpose of this review is to summarise current knowledge of the immunomodulatory functions of gonococcal PorB and to discuss the conflicting information on the properties of PorB to identify areas of interest for future research.

## Structure and function of gonococcal PorB

Gonococcal PorB is a homotrimeric outer-membrane porin, with each monomer ranging from 32 to 38 kDa and comprising 16 antiparallel β-strands with eight elongated extracellular loops [9]. Most of the loop regions extend into the extracellular space, although the longest loop, loop 3, is predicted to fold into the PorB barrel [9]. The PorB trimer is stabilised by the peptidoglycan binding protein RmpM (also known as PIII), with the N terminus of RmpM binding to the periplasmic face of PorB in a 1:3 ratio [9]. The N terminus of RmpM alone is sufficient to stabilise PorB complexes in *N. meningitidis* [10].

PorB facilitates ion exchange between the gonococcus and its external environment, with evidence that it is selective for the passage of anions [9,11]. The ionic conductance of PorB is voltage-dependent, with PorB from pathogenic *Neisseria* able to bind and translocate ATP, an interaction that modulates the voltage-dependence of PorB [11], and could influence inflammatory responses to infection by altering local ATP levels. The inward orientation of loop 3 and the presence of a protruding side chain from the β2-strand, known as a β-bulge, restrict the diameter of the PorB channel when compared with other 16-stranded bacterial porins [9,12]. The major role in acquisition of small molecules means that PorB is essential for the viability of *N. gonorrhoeae* [13], which is strongly constitutively expressed and does not undergo ON:OFF phase variation [14].

*N. gonorrhoeae* strains express a single PorB allele belonging to either the PorB.IA or PorB.IB class, which were historically distinguished both biochemically, through coagglutination assays and protease sensitivity, and immunologically, using specific monoclonal antibodies [15]. PorB.IA and PorB.IB can share up to 80% amino acid identity, with monomers of the former class tending to have a lower molecular weight [16]. Importantly, strains that express PorB.IA are more likely to disseminate and cause systemic gonococcal infection [17]. Both classes of gonococcal PorB also exhibit extensive genetic diversity, likely due to strong selective pressures from the host immune system, with genetic differences arising in both the transmembrane and exposed surface loops [18]. Indeed, a mutagenesis study revealed the majority of PorB amino acids are mutable, with only 20 residues considered essential [13]. Recent bioinformatic analysis of more than 5000 *N. gonorrhoeae* isolates found PorB the most diverse antigen, with 1229 different protein sequences amongst the strains [19].

## Gonococcal PorB and complement evasion

Gonococcal PorB has an important role in bacterial avoidance of recognition by the innate immune system, with some PorB proteins containing one or more domains that recruit soluble negative regulators of the complement system. In this way, *N. gonorrhoeae* can exhibit PorB-mediated serum resistance; however, many strains remain serum sensitive as they do not express a PorB that is able to bind negative regulators of complement. Complement is a powerful aspect of innate immunity that identifies invading pathogens. The complement system has three major arms: (i) the classical pathway, activated through antigen–antibody complexes, (ii) the lectin pathway, activated after deposition of lectin-based molecules on microbial surfaces, and (iii) the alternative pathway, which is constantly active at low levels, and acts as an amplification loop when the opsonin C3b binds to a foreign surface [20]. The activation of all pathways converges at the cleavage of C3, resulting in further C3b deposition and recruitment of complement factors C5–C9, which form the membrane attack complex and cause death by lysis [21]. Excessive complement activation is prevented by negative regulatory proteins, including C4b binding-protein (C4BP), factor H (FH), and the protease, factor I (FI) [21].

**Figure 1:**
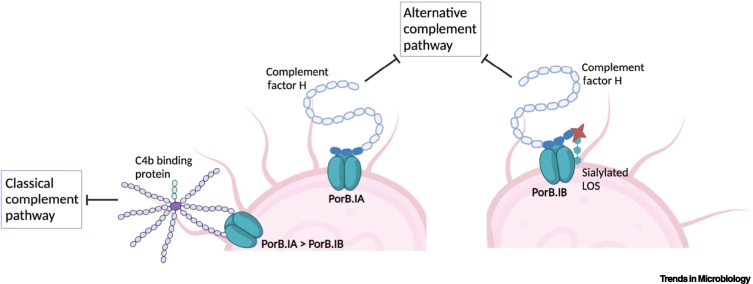
*Neisseria gonorrhoeae* PorB contributes to complement evasion. PorB recruits negative regulators of complement, C4b binding protein (C4BP), and factor H (FH), to block activation of the classical complement pathway and the alternative complement pathway, respectively. PorB.IA strains bind significantly more C4BP and FH than PorB.IB strains. PorB.IB binding to FH is increased in the presence of sialylated lipo-oligosaccharide (LOS). Figure created using BioRender.

*N. gonorrhoeae* is able to bind the negative regulator C4BP via PorB [22] (Figure 1). C4BP is a potent inhibitor of the classical and lectin pathways by directly binding to the opsonin C4b, and by acting as a cofactor for FI cleavage of C4b. C4BP prevents formation of the C4b–2a complex, a C3-convertase, hence preventing further cleavage of C3 into the chemoattractant C3a and opsonin C3b [23]. PorB.IA and PorB.IB differ in their ability to bind C4BP, with 90% of PorB.IA-expressing clinical isolates binding C4BP compared with only 19% of PorB.IB-expressing strains [24]. In efforts to determine the region responsible for C4BP recruitment, PorB.IA and PorB.IB again differed with loop 1 of PorB.IA (FA19) essential for C4BP binding, whereas loops 5 and 7 together formed a C4BP binding domain in PorB.IB (MS11) [22]. There is a significant association between the ability to bind C4BP and the evasion of complement activation by *N. gonorrhoeae* isolates [24].

Differences in gonococcal lipo-oligosaccharide (LOS) structure influence C4BP-PorB interaction. LOS is the major component of the outer membrane of *N. gonorrhoeae*, and is comprised of lipid A linked to saccharide branches [25]. The LOS layer is highly variable, and a single strain can vary its LOS due to phase variation [25]. Heptose I in gonococcal LOS can be linked to a glucose moiety as the first component of the LOS ⍺-chain if the strain expresses glycosyltransferase LgtF, and this proximal glucose is essential for C4BP binding to PorB in two PorB.IB expressing gonococcal strains (MS11 and 1291), but this was not mirrored in a PorB.IA-expressing strain (FA19) [26]. Loss of the phosphoethanolamine decoration of lipid A diminishes C4BP–PorB binding, but this is not influenced by whether a strain expresses PorB.IA or PorB.IB [27]. Overall, *N. gonorrhoeae* has evolved stable serum resistance through C4BP–PorB interaction, which appears to be an important characteristic in disseminating strains that express PorB.IA.

The sialylation of gonococcal LOS is well documented in its ability to promote recruitment of FH to the gonococcal surface, conferring serum resistance by increasing FI cleavage of the C3b opsonin deposited on the bacterial surface, generating inactive C3b (iC3b) [28]. FH binding can occur independently of LOS sialylation by interacting with PorB [29] (Figure 1). PorB.IA-expressing strains closely associated with disseminated gonococcal infections are more often serum resistant than PorB.IB-expressing strains, which are primarily associated with genital-tract infection [30]. Indeed, non-sialylated *N. gonorrhoeae* strains expressing PorB.IA recruited more FH to their surface than PorB.IB strains, contributing to serum resistance during disseminated gonococcal infections [29]. The site of FH binding was narrowed down to loop 5 of PorB.IA [29,31], where loops 3 to 7 are involved in PorB.IB (FA1090) binding to FH [32]. LOS sialylation increases the binding of FH to serum-resistant PorB.IB (F62 and MS11) expressing strains [33], suggesting a tripartite interaction between PorB.IB, LOS sialic acid and FH [34]. However, this triad may act as an Achilles heel for *N. gonorrhoeae*, as it is unable to distinguish FH from complement FH-related protein 5 (CFHR5), where the latter enhances complement-mediated lysis of the bacterium [35]. Overall, the ability of PorB to recruit the negative complement regulator FH is important for gonococcal survival as PorB is constantly expressed at high levels, while LOS expression is phase variable. Equally, PorB.IA and sialylated PorB.IB strains contribute specifically to human complement resistance, and not to complement from other species, suggesting a role in the species-specific restriction of *N. gonorrhoeae* [36].

Some antibodies, termed ‘blocking antibodies’, bind to bacterial surface antigens and prevent complement-dependent killing by preventing the binding of bactericidal antibodies that would trigger the complement cascade [34]. For *N. gonorrhoeae*, IgG antibodies isolated from non-bactericidal sera blocked killing of the bacterium [37]. The primary target of the blocking antibodies is thought to be the outer-membrane protein RmpM, which stabilises trimeric PorB [9,38]. Both immunisation with gonococcal RmpM and passive transfer of RmpM-specific IgGs reduced the efficacy of an anti-LOS monoclonal antibody that protects mice from *N. gonorrhoeae* infection by increasing C3 deposition onto the bacterial surface [39]. Further, women who have circulating anti-RmpM antibodies have increased susceptibility to gonococcal infections [40]. Although RmpM elicits blocking antibodies and forms a complex with PorB, thus far there is no evidence of blocking antibodies being directed against PorB. However, anti-RmpM antibodies did reduce the binding of PorB-specific monoclonal antibodies [39].

## Innate immune responses to neisserial PorB

Upon infection, aspects of the gonococcal surface, including PorB, are recognised by Toll-like receptors (TLRs) expressed on the surface of innate immune cells, such as dendritic cells (DCs) and macrophages, as well as non-immune cells, such as epithelial cells [41]. TLRs are a key component of the innate immune system owing to their ability to recognise conserved microbial structures known as pathogen-associated molecular patterns (PAMPs) [41]. TLR2 is involved in the recognition of bacterial membrane lipoproteins, Gram-positive bacteria, and mycobacterial species [42]. TLR4, alongside its coreceptor CD14 [43], plays a central role in the recognition of LOS/lipopolysaccharide (LPS), the endotoxin on the surface of Gram-negative bacteria. Engagement of TLRs signals members of the nuclear factor-κB family of transcription factors to translocate to the nucleus, inducing transcription of proinflammatory cytokines and chemokines, and other inflammatory mediators [44]. For *Neisseria* spp., several studies have described TLR2 recognition of PorB [45., 46., 47.].

**Figure 2:**
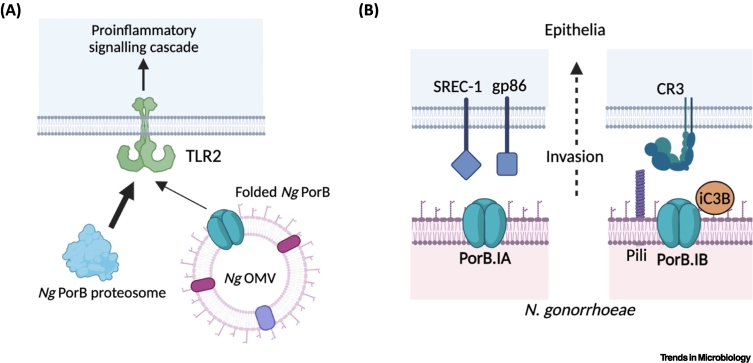
*Neisseria gonorrhoeae* PorB interacts with numerous host cell surface proteins. (A) Gonococcal PorB prepared as protein aggregates (proteosomes) stimulates significant proinflammatory signalling response via Toll-like receptor 2 (TLR2) activation, but folded PorB only activates TLR2 to a modest degree. (B) PorB facilitates invasion of epithelial cells. PorB.IA alone interacts with a scavenger receptor on endothelial cells-1 (SREC-1) and human heat shock glycoprotein 86 (gp86) to facilitate invasion and transcytosis of epithelia. PorB.IB requires pili and inactive C3b (iC3b) to interact with complement receptor 3 (CR3) to enable invasion. Figure created using BioRender. Abbreviations: *Ng*, *Neisseria gonorrhoeae*; OMV, outer-membrane vesicle.

PorB from *N. meningitidis* has been used in numerous studies as an adjuvant to enhance immune responses to poorly immunogenic antigens, occurring via TLR2 signalling [45,46]. The vast majority of studies that discuss ‘neisserial PorB’ as an immune adjuvant are more specifically studying *N. meningitidis* PorB, with little information on *N. gonorrhoeae* PorB-TLR2 signalling. A recent study did confirm that *N. gonorrhoeae* PorB.IB (from strain FA1090) can trigger the TLR2 signalling cascade [47] (Figure 2A). However, this activation was shown to be heavily reliant upon the presentation method of PorB. TLR2 signalling was increased sevenfold in response to *N. gonorrhoeae* PorB.IB (FA1090) proteosomes, which are protein aggregates formed when detergent is removed during the protein purification process [48], as used in immunopotentiation studies for *N. meningitidis* PorB [45., 46., 47.]. Recombinant gonococcal PorB.IB refolded into micelles using detergent that was more similar to the native presentation of PorB trimers in the gonococcal outer membrane or in OMVs, elicited a more modest TLR2 activation, which increased only 1.5-fold [47]. Together, this suggests that PorB presented during infection may confer different properties to PorB studied as protein aggregates. However, meningococcal PorB proteosomes that activated TLR2 signalling were purified directly from *N. meningitidis*, whereas refolded gonococcal PorB.IB within micelles were produced recombinantly in *Escherichia coli* [47,49]. The method of production of PorB may also influence the conclusions made on the immunostimulatory properties of neisserial porins.

*N. gonorrhoeae* PorB has been targeted for use in conjunction with bacterial ghosts as a vaccine. Bacterial ghosts are empty Gram-negative cells [50] that can serve as a nondenatured representation of a bacterial surface for vaccine use [51], and can also be used as a delivery system [50,51]. *Salmonella enteritidis* (SE) ghosts were used to carry DNA encoding *N. gonorrhoeae* PorB (WHO-A), and the presence of *porB* DNA was found to increase *Salmonella*-specific antibodies and enhance protection against *Salmonella* in mice, compared with SE ghosts alone [52]. Further, SE ghosts delivering *porB*-encoding DNA elicited higher levels of PorB-specific serum antibodies than PorB-encoding DNA alone [53,54]. In the bacterial ghost system it appears that *N. gonorrhoeae* PorB acts as an immune stimulant, however the specific mechanism remains unexplored. Neisserial PorB is immunogenic and elicits production of specific antibodies, observed during natural infection [55,56]. Immunisation with the meningococcal 4CMenB vaccine, which contains PorB within OMVs, elicits bactericidal antibodies specific to meningococcal PorB [57]. Further, 4CMenB immunisation generates cross-protection against gonococcal infection in mice, with PorB identified as a cross-reactive antigen [58].

## Invasion of host cells by *N. gonorrhoeae*

PorB participates in the adhesion to and invasion of epithelial cells by *N. gonorrhoeae*. Intracellular pathogens evade host immune surveillance by surviving within cells, a process that requires adhesion, invasion, avoidance of host intracellular killing mechanisms, and modulation of the cellular environment to access nutrients [59]. *N. gonorrhoeae* can reside within epithelial cells, macrophages, and neutrophils [60]. Pili, opacity-associated proteins (Opa), LOS, and PorB all act as adhesins, allowing the gonococcus to interact with a variety of cell types [61]. *N. gonorrhoeae* primarily colonises mucosal epithelial cells and is able to invade and transcytose monolayers of epithelial cells, which is thought to contribute to disseminated gonococcal infection [59].

Gonococcal strains expressing PorB.IA, associated with disseminated gonococcal infection, are able to invade epithelial cells and allelic replacement with PorB.IB abrogates the invasive phenotype, exemplified by interchange of MS11 PorB.IB and FA19 PorB.IA [62]. Further, PorB.IA-mediated (MS11 derivative N927) invasion was enhanced in the presence of human serum, but the exact mechanism for this is unknown [59]. Invasion of epithelial cells that is mediated by Opa_50_ binding to heparan sulfate-containing proteoglycans (HSPGs) was impaired after the deletion of loop 1 of PorB.IB in strain MS11 [62,63]. Equally, when PorB.IB was replaced with *Neisseria lactamica* PorB, the strains were unable to induce efficient Opa_50_-mediated uptake by epithelial cells [62]. Several host surface molecules have been linked to PorB-mediated gonococcal invasion of epithelial cells (Figure 2B). Complement receptor 3 (CR3) is present on female genital-tract epithelia and is a promiscuous receptor able to bind numerous ligands [64,65]. Gonococcal PorB is able to bind to CR3; however, in this case, the interaction is independent of the PorB.IA/PorB.IB classification [65]. Pili and iC3b on the gonococcal surface, the latter deposited following complement activation, also bind to CR3. Along with PorB.IB (FA1090), these three components form a co-operative mechanism for adhering to and invading primary cervical epithelial cells, as opposed to PorB alone [65]. For PorB.IA-mediated invasion, where PorB acts alone, human heat shock glycoprotein 86 and SREC-1 (scavenger receptor expressed by endothelial cell-1) are the binding sites for PorB.IA (MS11 derivative N927) on epithelial cells, with the latter being key for bacterial uptake [66,67]. Overall, PorB is involved in the ability of *N. gonorrhoeae* to adhere to epithelial cells, enabling invasion and intracellular survival. PorB.IA is instrumental in the invasion and transcytosis of epithelia, which significantly contributes to the dissemination of PorB.IA-expressing strains.

## *N. gonorrhoeae* PorB modulates innate immune cells

*N. gonorrhoeae* engages sialic acid recognising Ig-like lectins (Siglecs) to impair immune cell activation through LOS sialic acid and via PorB if LOS is unsialylated, with PorB.IA and PorB.IB exhibiting similar binding [68]. Siglecs are primarily expressed by innate immune cells and serve to recognise ‘self’ sialic acid patterns and deliver inhibitory signals to prevent proinflammatory responses [68]. A potential conserved binding motif in PorB serves to downregulate proinflammatory responses by innate immune cells. Gonococcal PorB is stably expressed, thereby giving the bacterium an alternative method to dampen host proinflammatory responses when LOS undergoes phase variation, or when there is no source of sialic acid available for sialylation.

**Figure 3:**
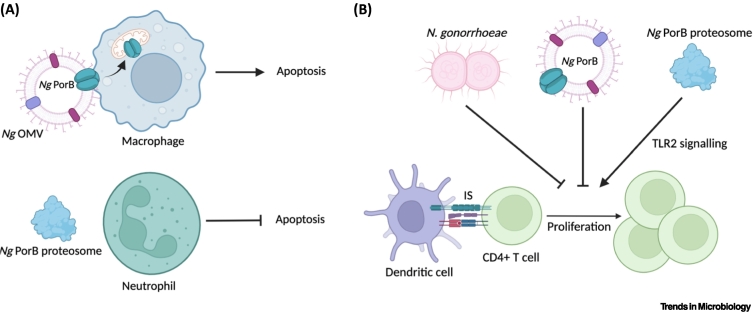
*Neisseria gonorrhoeae* PorB influences innate and adaptive immune responses. (A) Folded gonococcal PorB translocates to the mitochondria of macrophages and causes apoptosis, whereas PorB proteosomes inhibit apoptosis in neutrophils. (B) Whole gonococci and folded PorB suppress the ability of dendritic cells to stimulate CD4^+^ T cell proliferation, whereas PorB proteosomes promote Toll-like receptor 2 (TLR2) signalling. Figure created using BioRender. Abbreviations: IS, immunological synapse; *Ng*, *Neisseria gonorrhoeae*; OMV, outer-membrane vesicle.

*N. gonorrhoeae* is able to survive within macrophages and impede apoptosis, thereby promoting their intracellular survival [69]. Microorganisms engulfed by macrophages enter a phagosome that sequentially acquires different proteins during maturation, eventually fusing with acidic lysosomes to digest the engulfed material [70]. Purified gonococcal PorB.IA (VP1) arrests phagosome maturation in macrophages, which is consistent with a lack of colocalisation of *N. gonorrhoeae* and lysosomal markers within macrophages [69,71]. PorB.IB (MS11-A) introduced to macrophages in its native conformation from *N. gonorrhoeae* OMVs colocalises with mitochondria and induces apoptosis [72] (Figure 3A). This phenomenon appears to differ for *N. meningitidis*, where delivery of meningococcal PorB to the mitochondria protected host cells from apoptosis [73., 74., 75.]. For *N. gonorrhoeae*, it is likely that additional factors contribute to the modulation of macrophage apoptosis as whole gonococci had different effects on different human macrophage cell lines, inducing apoptosis in THP-1 cells, but preventing apoptosis in U937 cells and primary human macrophages [69,76].

*N. gonorrhoeae* infection leads to an influx of neutrophils into the genital tract, with gonococcal PorB contributing to the manipulation of neutrophils to enable bacterial survival. *N. gonorrhoeae* selectively prolongs the survival of neutrophils by preventing apoptosis, allowing the bacterium to persist intracellularly, where PorB contributes to the inhibition of neutrophil killing mechanisms [77] (Figure 3A). Neutrophils use oxidative and non-oxidative pathways to kill invading pathogens, with the latter involving the release of antimicrobial proteases from intracellular vesicles, in a process termed degranulation [78]. Purified gonococcal PorB inhibits degranulation (strain R10) and the production of reactive oxygen species by neutrophils (strain VP1) [79., 80., 81.]. In gonococcal strains expressing PorB.IB (FA1090) that can bind C4BP (described above), the recruitment of C4BP reduced the ability of neutrophils to phagocytose the bacterium, alongside suppressing production of reactive oxygen species [82]. PorB.IB (in strain Pgh 3-2) also inhibits actin polymerisation in activated neutrophils and reduces the expression of complement receptors, thereby reducing phagocytosis [80]. Interestingly, *N. meningitidis* lacking both of its major outer membrane porins, PorA and PorB, was still able to inhibit apoptosis and the oxidative burst in human neutrophils [83]. Purified PorB protein was primarily used to examine gonococcal PorB interaction with neutrophils [77., 78., 79., 80., 81.,^83^], however the effects of refolded PorB in its native conformation have not been described, therefore the precise effects of gonococcal PorB still need to be defined.

## *N. gonorrhoeae* PorB influences the adaptive immune response

During natural infection *N. gonorrhoeae* suppresses T helper type (Th)1/Th2 adaptive immune responses, instead priming a Th17-driven innate immune response to promote its survival [84]. Further, *N. gonorrhoeae* can suppress the ability of DCs to induce CD4^+^ T cell proliferation, demonstrating modulation of the protective immune response at the level of antigen presentation [85]. DC induction of T cell proliferation was also suppressed by the presence of OMVs, with PorB.IB (FA1090) playing a key role [47]. Treatment of DCs with refolded PorB.IB (FA1090), as it would occur during infection and in OMVs, was responsible for the immunosuppression of DCs [47] (Figure 3B). By contrast, PorB.IB proteosomes did not exhibit immunosuppressive properties and were able to stimulate TLR2 responses, as outlined earlier [47] (Figure 3B). Together this suggests that gonococcal PorB in its native folded conformation plays a key role in the suppression of adaptive immune responses.

## Concluding remarks and future perspectives

*N. gonorrhoeae* is well adapted to its human host and promotes its survival by manipulating host immune responses. PorB is the most abundant outer-membrane protein of the gonococcus and is essential for survival of the bacterium. Therefore, perhaps unsurprisingly, PorB has multiple roles during infection, including contributing significantly to immunosuppression. Gonococcal PorB represses the killing mechanisms of neutrophils and macrophages, contributes to complement evasion, facilitates invasion of host cells, and suppresses the adaptive immune response at the antigen presenting cell level. There is conflicting evidence on gonococcal PorB modulation of apoptosis, both inducing and inhibiting apoptosis in epithelial cells and macrophages, suggesting that specific cell type may also influence the effects of PorB. The different modulation of apoptosis in macrophages and epithelial cells by PorB may well be important over the course of *N. gonorrhoeae* infection (see Outstanding questions), and the implications of this would be hugely beneficial for understanding gonococcal pathogenesis, disease progression, and for vaccine development.

There appear to be contrasting properties of PorB from *N. gonorrhoeae*, *N. meningitidis*, and commensal *Neisseria* species. Therefore, it is important to not assume all attributes apply to all *Neisseria* porins. In addition, the neisserial *porB* gene is hypervariable with frequent intra- and inter-species recombination events known to occur [86], leading to constant diversification of PorB that is also likely to influence its immunomodulatory properties. The purification methods for studying PorB in isolation appear to significantly contribute to conclusions about the properties of the porin. It is likely that refolded recombinant PorB more accurately reflects the presentation of PorB to the immune system during infection, and thus should be considered in future studies. Equally, due consideration should be given to whether PorB was purified in its native form directly from *Neisseria* spp., or recombinantly produced and refolded, as this may also influence conclusions on the properties of neisserial PorB. Understanding the differences in immunomodulatory properties of PorB from different Neisseria species would be beneficial for vaccine development, particularly as meningococcal PorB does not appear to hamper the success of OMV-based meningococcal vaccines. Any observed differences could lead to identification of the region(s) responsible for immune modulation by gonococcal PorB, and therefore potential mutagenesis to nullify the immunosuppressive properties of this essential porin (see Outstanding questions). Indeed, the high abundance of PorB in gonococcal OMVs conferring immunosuppressive properties represents an important consideration for the development of a successful OMV-based vaccine against this bacterium.

Outstanding questions To what extent do the immunomodulatory properties of PorB from different species of *Neisseria* vary?

Which region(s) of gonococcal PorB are critical for immunomodulation?

Does PorB have differential effects on different immune cells to enable survival of bacteria during gonococcal infection?

How will the presence of immunomodulatory PorB in high abundance affect the success of a gonococcal outer-membrane-vesicle-based vaccines?

Alt-text: Outstanding questions

## Declaration of interests

R.J. and C.T. are inventors on a patent for developing gonococcal vaccines.
